# Supplementary material for: Assembly of “3D” plasmonic clusters by “2D” AFM nanomanipulation of highly uniform and smooth gold nanospheres
Source: Sci Rep. 2017 Jul 20;7:6045. doi: 10.1038/s41598-017-06456-w (PMC5519739; doi:10.1038/s41598-017-06456-w)
Supplement: Supplementary file 1 — Supplementary Information [file 41598_2017_6456_MOESM1_ESM.pdf]

Supplementary Information *for*

Assembly of "3D" plasmonic clusters by "2D" AFM manipulation of highly uniform and smooth gold nanospheres

Kyung Jin Park[1,+], *Ji-Hyeok Huh*[1,+], *Dae-Woong Jung*[2], *Jin-Sung Park*[3], *Gwan H. Choi*[2], *Gaehang Lee*[4], *Pil J. Yoo*[2], *Hong-Gyu Park*[3], *Gi-Ra Yi*[2], and *Seungwoo Lee*[1,2*]

[1] SKKU Advanced Institute of Nanotechnology (SAINT), Sungkyunkwan University (SKKU), Suwon 16419, Republic of Korea

[2] School of Chemical Engineering, Sungkyunkwan University (SKKU), Suwon 16419, Republic of Korea

[3] Department of Physics, Korea University, Seoul 02841, Republic of Korea

[4] Korea Basic Science Institute (KBSI) and University of Science and Technology, Daejeon 34113, Republic of Korea

[+]Equally contributed to this work

*Email: seungwoo@skku.edu

Contents:

1. Fabrication of nanohole template: Rigiflex nanoimprint lithography
2. Uniformity and crystallinity of gold nanospheres (AuNSs)
3. Dry-transfer printing of AuNSs onto the top surface of PMMA nanohole template
4. Effect of PMMA nanohole on resonance modes
5. Optical resonance of trimer, when illuminated with *p*-polarized light
6. Optical properties of symmetric tetrahedral cluster for *p*-pol illumination
7. Optical properties of asymmetric tetrahedral cluster

1. Fabrication of nanohole template: Rigiflex nanoimprint lithography

The silicon (Si) master of nanohole template, shown in Figure S1a, was developed by electron beam (e-beam) lithography, combined with reactive ion etching (RIE). Poly(methylmethacrylate) (PMMA) was patterned by a controlled e-beam writing and subsequent etching; then, the exposed Si area was selectively carved using RIE. Then, rigid mold made of poly(urethane) (PUA) was replicated and used to imprint PMMA (i.e., rigiflex nanoimprint lithography).[S1] Figure S1b and S1c respectively display optical microscope images (dark-field mode) of PUA mold and PMMA nanohole template.

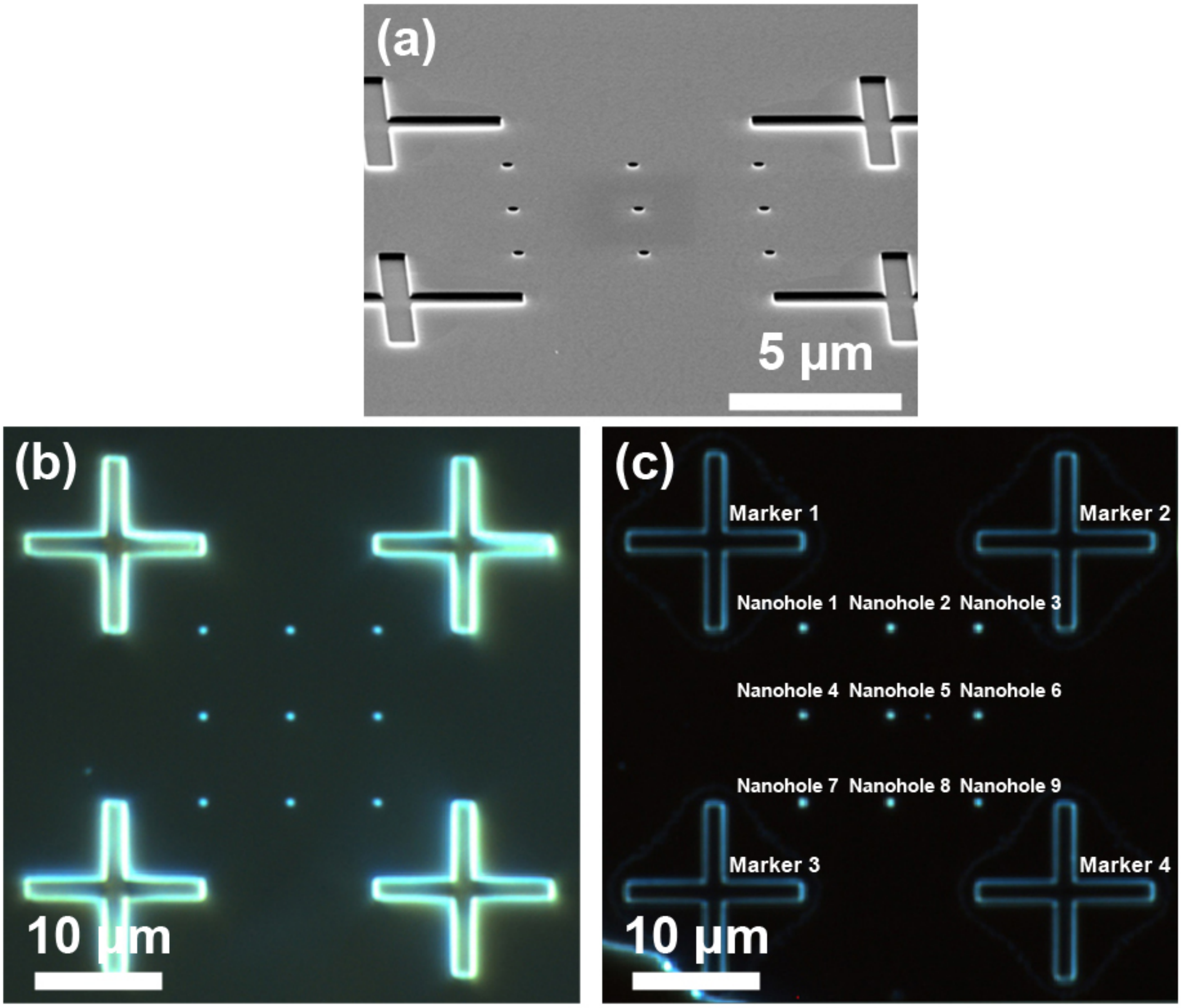

Figure S1. (a) Scanning electron microscopy (SEM) image of silicon (Si) nanohole master pattern, which was developed by electron-beam (e-beam) lithography and reactive ion etching (RIE). (b) Optical microscopy image (dark-field mode) of polyuthethane (PUA) mold replicated from Si master. (c) Optical microscopy image (dark-field mode) of the polymer nanohole pattern, which was obtained by PUA mold-enabled nanoimprinting onto a flat polymethylmethacrylate (PMMA) thin film.

2. Uniformity and crystallinity of gold nanospheres (AuNSs)

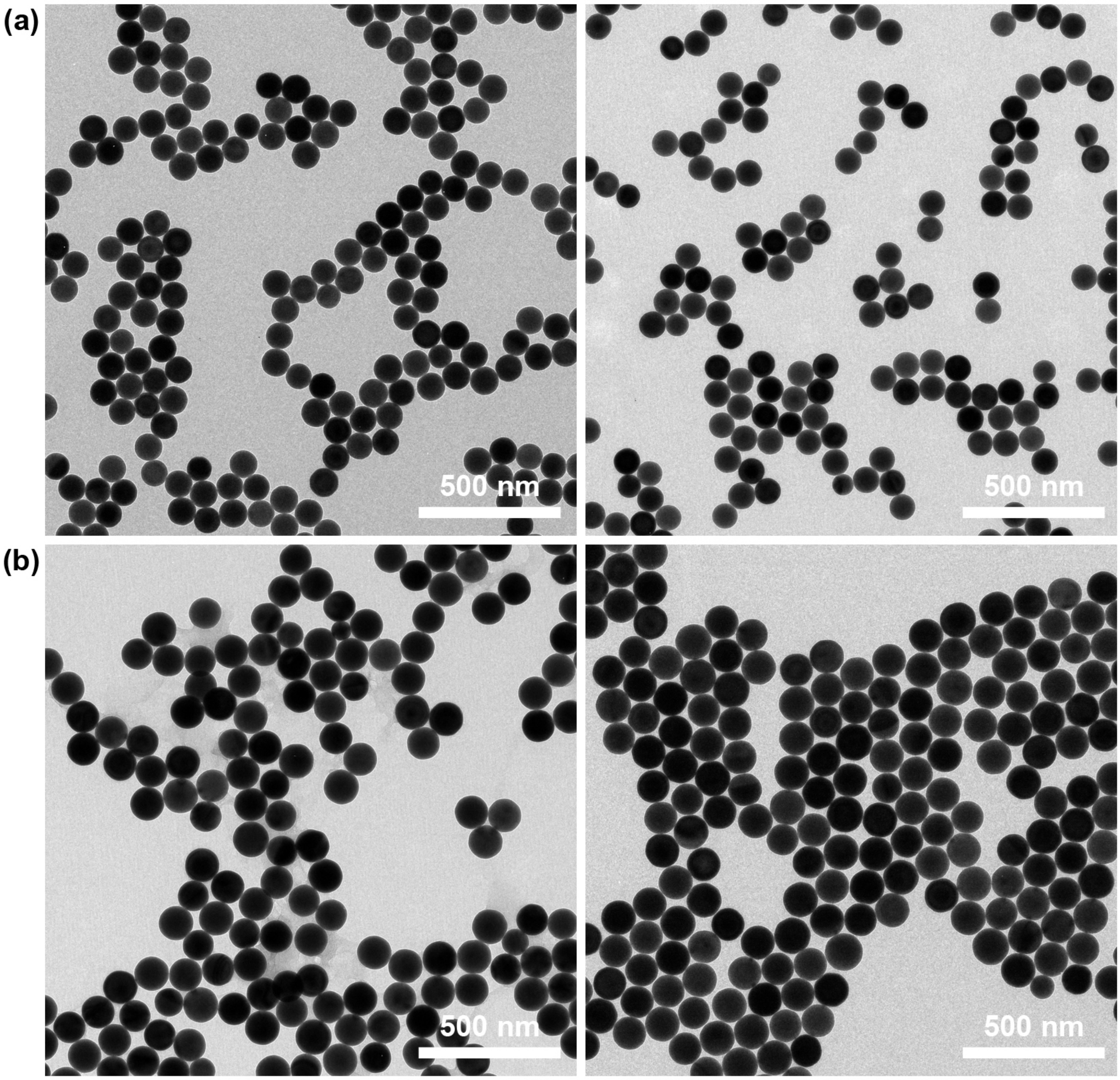

Figure S2. Transmission electron microscopy (TEM) images of (a) 77 nm and (b) 100 nm AuNSs used in this work.

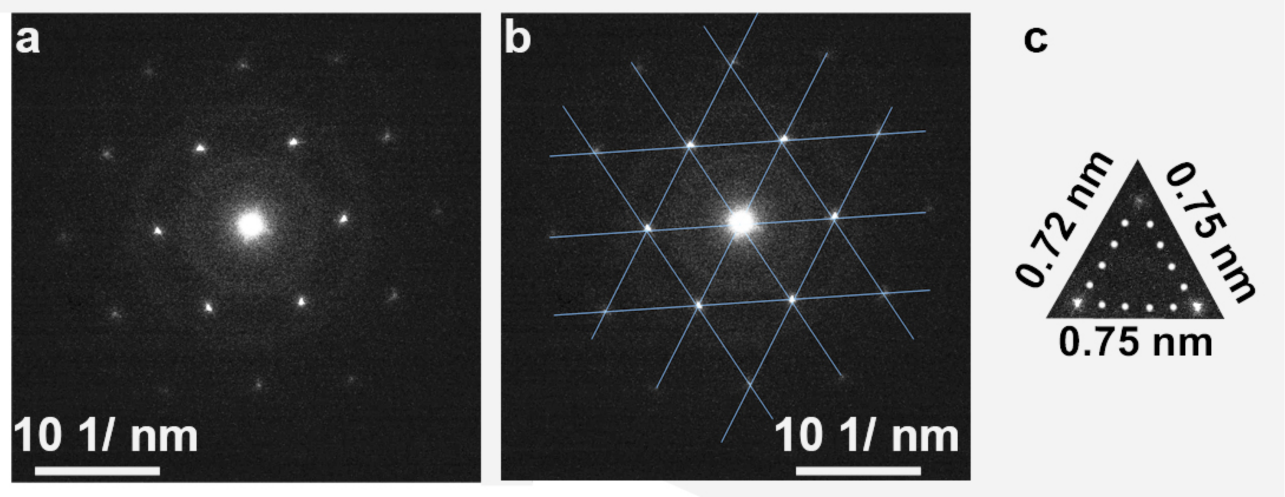

Figure S3. Diffraction pattern analysis of AuNSs used in this work.

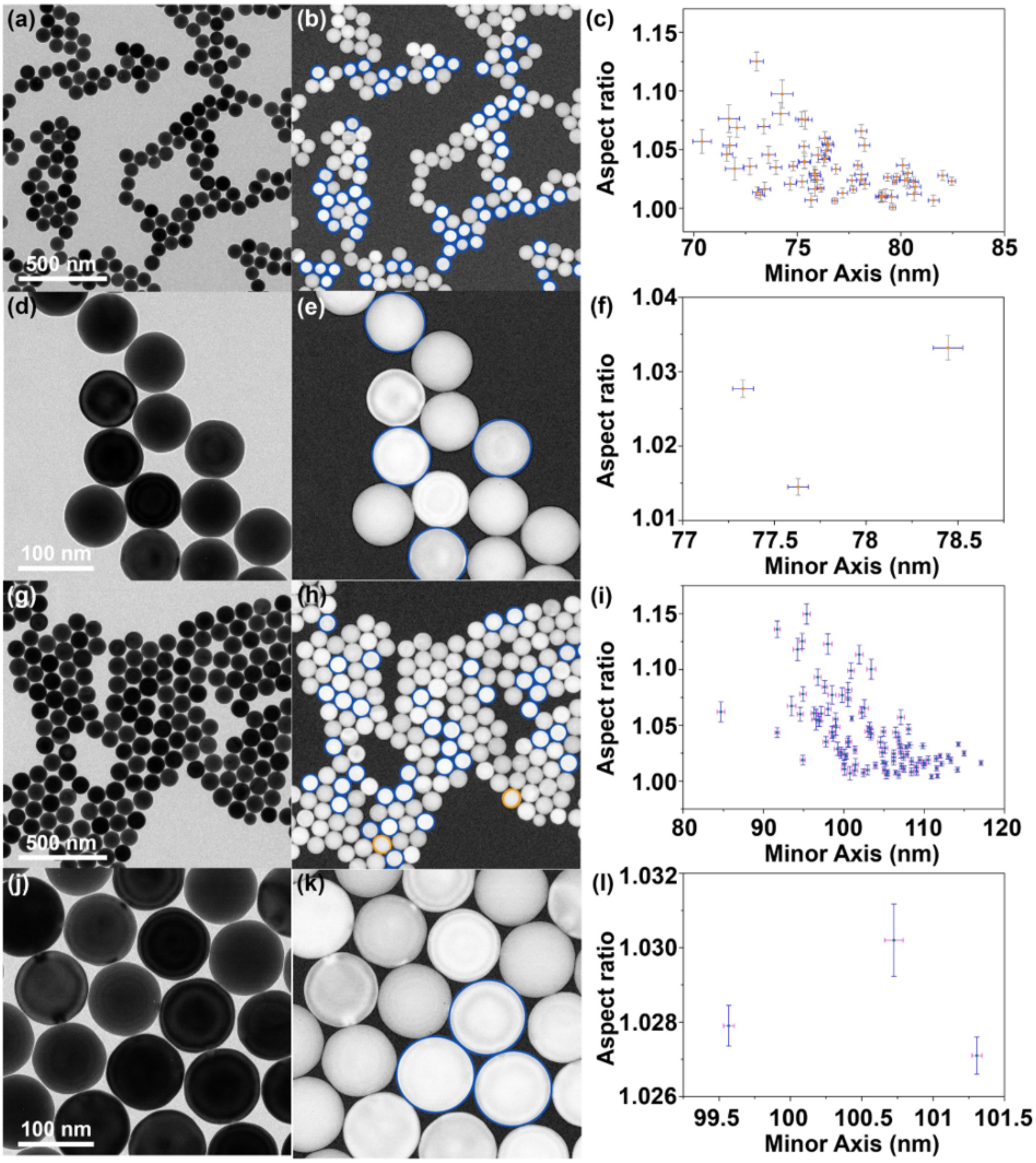

Figure S4. Algorithmic analysis of our AuNSs' uniformity using C. Mirkin's method.[S2] (a) Original TEM image and (b) analyzed TEM image of 77 nm AuNSs taken at a relatively low magnification. (c) Analyzed aspect ratio (i.e., the ratio of minor axis to major axis) based on (b). (d) Original TEM image and (e) analyzed TEM image of 77 nm AuNSs taken at a relatively high magnification. (f) Analyzed aspect ratio based on (e). (g) Original TEM image and (h) analyzed TEM image of 100 nm AuNSs taken at a relatively low magnification. (i) Analyzed aspect ratio (i.e., the ratio of minor axis to major axis) based on (h). (j) Original TEM image and (k) analyzed TEM image of 100 nm AuNSs taken at a relatively high magnification. (l) Analyzed aspect ratio based on (k). The blue and orange circles, shown in (b), (e), (h), and (k), respectively highlight the near unity and relatively high aspect ratio of AuNSs.

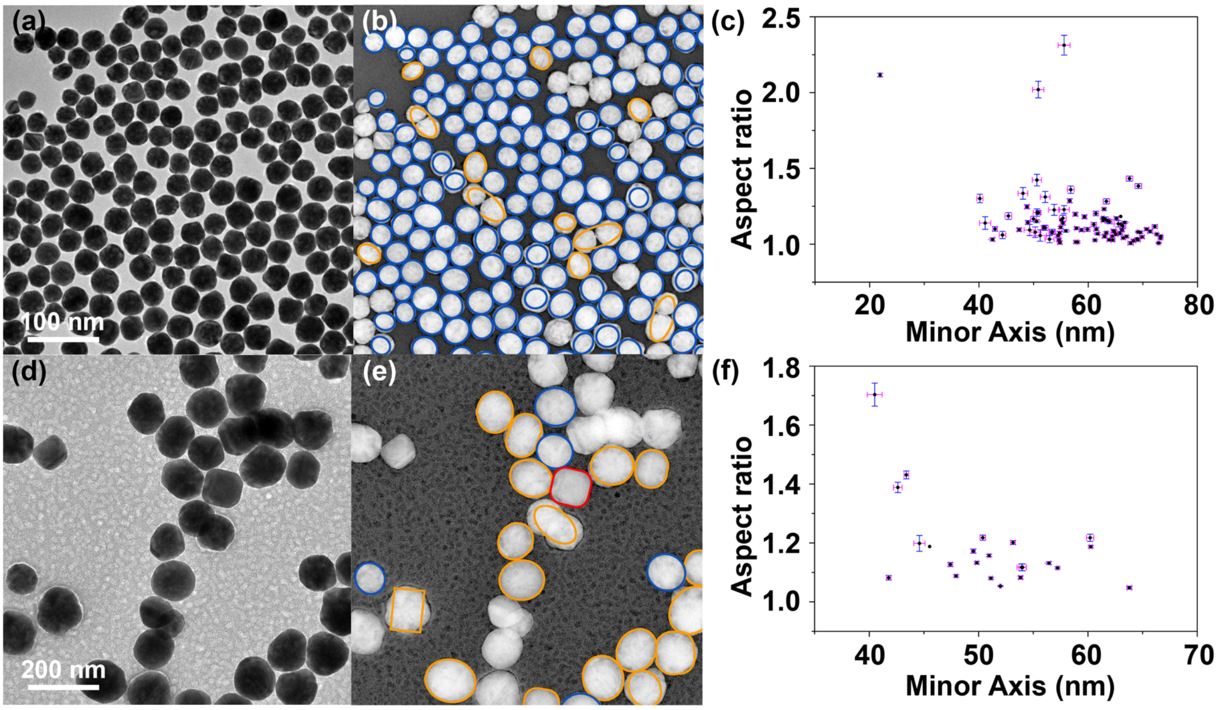

Figure S5. Algorithmic analysis of the polygonal shaped AuNSs (~ 55 nm size), which were synthesized by conventional seed-growth method.[S3] (a) Original and (b) analyzed TEM image taken at a relatively low magnification. (c) Analyzed aspect ratio (i.e., the ratio of minor axis to major axis) based on (b). (d) Original and (e) analyzed TEM image taken at a relatively high magnification. (f) Analyzed aspect ratio based on (e). The blue and orange circles, shown in (b) and (e), respectively highlight the relatively low and high aspect ratio. Red box, presented in (e), indicates the shape quite close to cube.

3. Dry-transfer printing of AuNSs onto the top surface of PMMA nanohole template

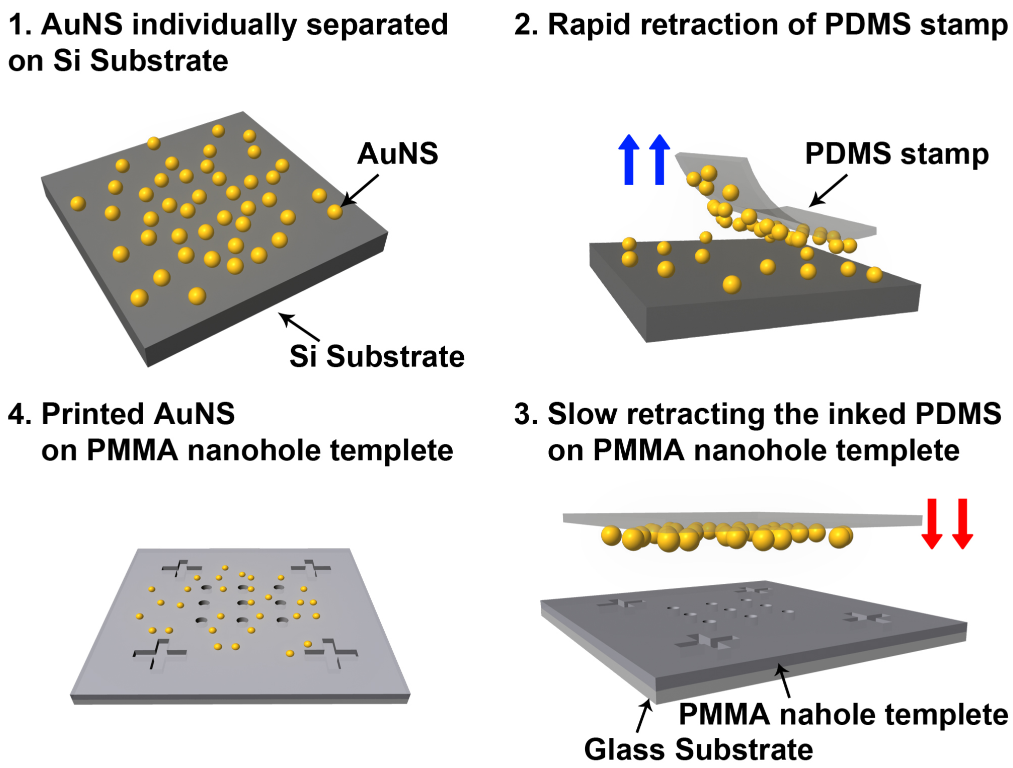

Figure S6. Schematic representation of dry transfer printing process.

4. Effect of PMMA nanohole on resonance modes

In this section, we theoretically verified the influence of nanohole substrate on the resonance behavior of plasmonic metamolecules (e.g., in this simulation, trimer consisting of equivalent 77 nm AuNSs was designed to be placed nearby the wall of PMMA nanohole as with the actual experiment). Compared with a flat glass substrate, PMMA nanohole could shift and change the resonance peak, as shown in Figure S7. However, the important physical characteristics of trimer were found to be almost similar between a flat glass and PMMA nanohole.

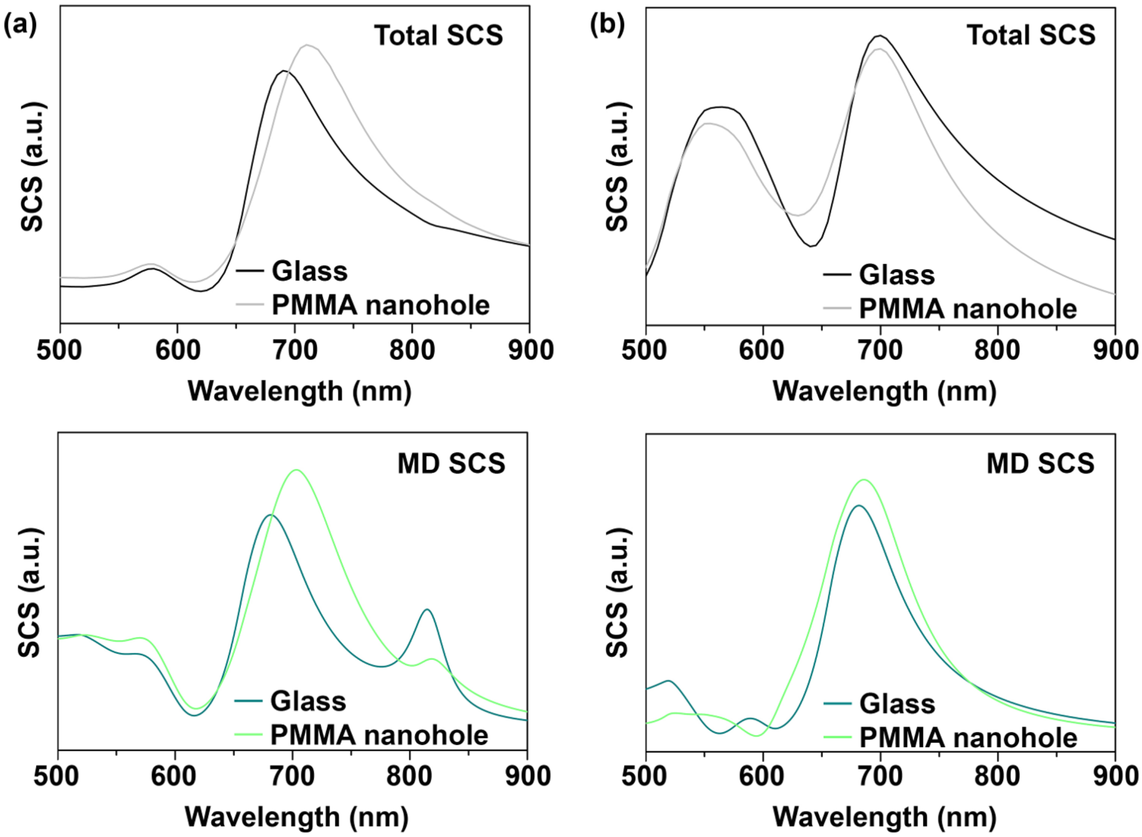

Figure S7. The numerically simulated scattering cross section (SCS) of trimer for (a) *s*-pol and (b) *p*-pol. Here, trimer was placed onto a flat glass substrate and within PMMA nanohole template. Electric dipole (ED) is not included in this analysis, as total SCS is mainly determined by ED. It was clearly observed that magnetic dipole (MD) resulting from a circulating current displacement cannot be induced for *p*-pol illumination (810 nm).

5. Optical resonance of trimer, when illuminated with *p*-polarized light

Figures S8a-d show several representative dark-field optical microscope (DFOM) images of the assembled trimers; uniformly distributed yellowish scattering colors were clearly observed. Atomic force microscope (AFM) images of the trimer, assembled within PMMA nanohole, is shown in Figure S8e. The triangular geometry made of three 77 nm AuNSs was confirmed; these three AuNSs were close-packed with 1.5 nm gap.

Figures S8f-m demonstrate numerically predicted and experimentally measured scattering spectra of the assembled trimer. When *p*-polarized light was illuminated with a 64° slant angle, the circulating displacement current and electric field along the planar ring motif of trimer cannot be induced. This means that two main scattering peaks, shown in Figure S8f, originated from electric dipole (ED) (at 700 nm) and electric quadrupole (EQ) (at 550 nm) (see Figure S9). The total scattering cross section (SCS) is mainly determined by ED and EQ. Experimental measurement, presented in Figure 4h, matched well with such theoretical prediction (Figure S8h); probing the high structural quality of the assembled trimer.

For *s*-pol illumination (Figure S8i-j), the circulating displacement current and electric field can be induced along the planar ring motif of trimer, so as to activate the MD resonance at 815 nm (i.e., optical magnetism). Both the circulating electric field (top panel in Figure S8i) and the resultant magnetic field (bottom panel of Figure S8i) confirmed the presence of optical magnetism. The broad peak at 610 nm to 800 nm in Figure S8j originated from ED (see Figure S8j). Due to their weakness, the MD mode cannot be not obvious in the total SCS (Figure S8i). Actually, in dark-field spectroscopy without cross-analyzer, the MD resonance at 820 nm was not elusive, as expected (see Figure S8l). After the insertion of cross-analyzer, the MD resonance become more clear. Even if its peak wavelength was slightly red-shifted, the important features of dark-field scattering well matched with theoretical predictions. The extremely precise nanomanipulation of AFM tip was repeated until the desired scattering spectra was obtained.

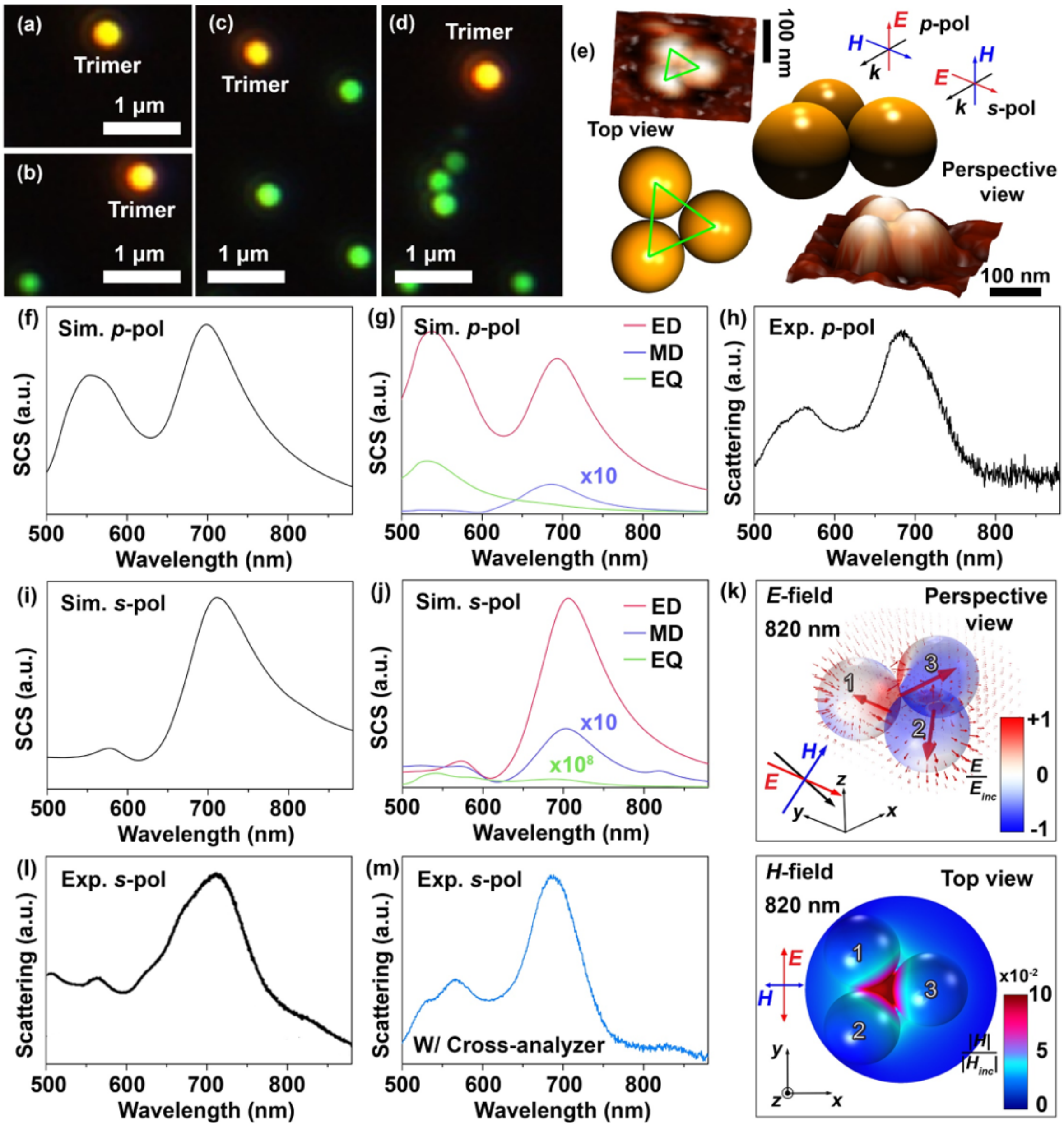

Figure S8. (a-d) Dark-field optical microscope (DFOM) images of several trimer, assembled via AFM nanomanipulation of equivalent 77 nm AuNSs. (e) 2D and 3D Atomic force microscope (AFM) image of the trimer, assembled within PMMA nanohole. Triangular geometry was confirmed. (f-g) Numerically simulated SCS of trimer for *p*-pol: (f) total SCS and (g) ED/MD/electric quadrupole (EQ) SCS. (h) Dark-field scattering spectrum of trimer for *p*-pol. (i-j) Numerically simulated SCS of trimer for *s*-pol illumination: (i) total SCS and (j) ED/MD/QD SCS. (k) Spatial distribution of electric field and magnetic field intensity at 820 nm for *s*-pol. (l-m) Dark-field scattering spectrum of trimer for *s*-pol: (l) without cross-analyzer and (m) with cross-analyzer.

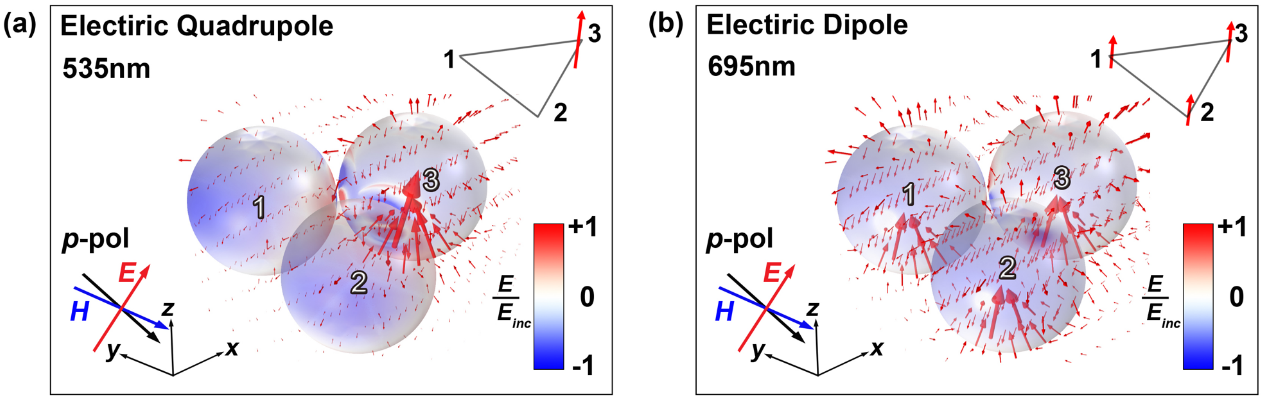

Figure S9. The characteristics of (a) ED and (b) EQ resonances of trimer for *p*-pol.

6. Optical properties of symmetric tetrahedral cluster for *p*-pol illumination

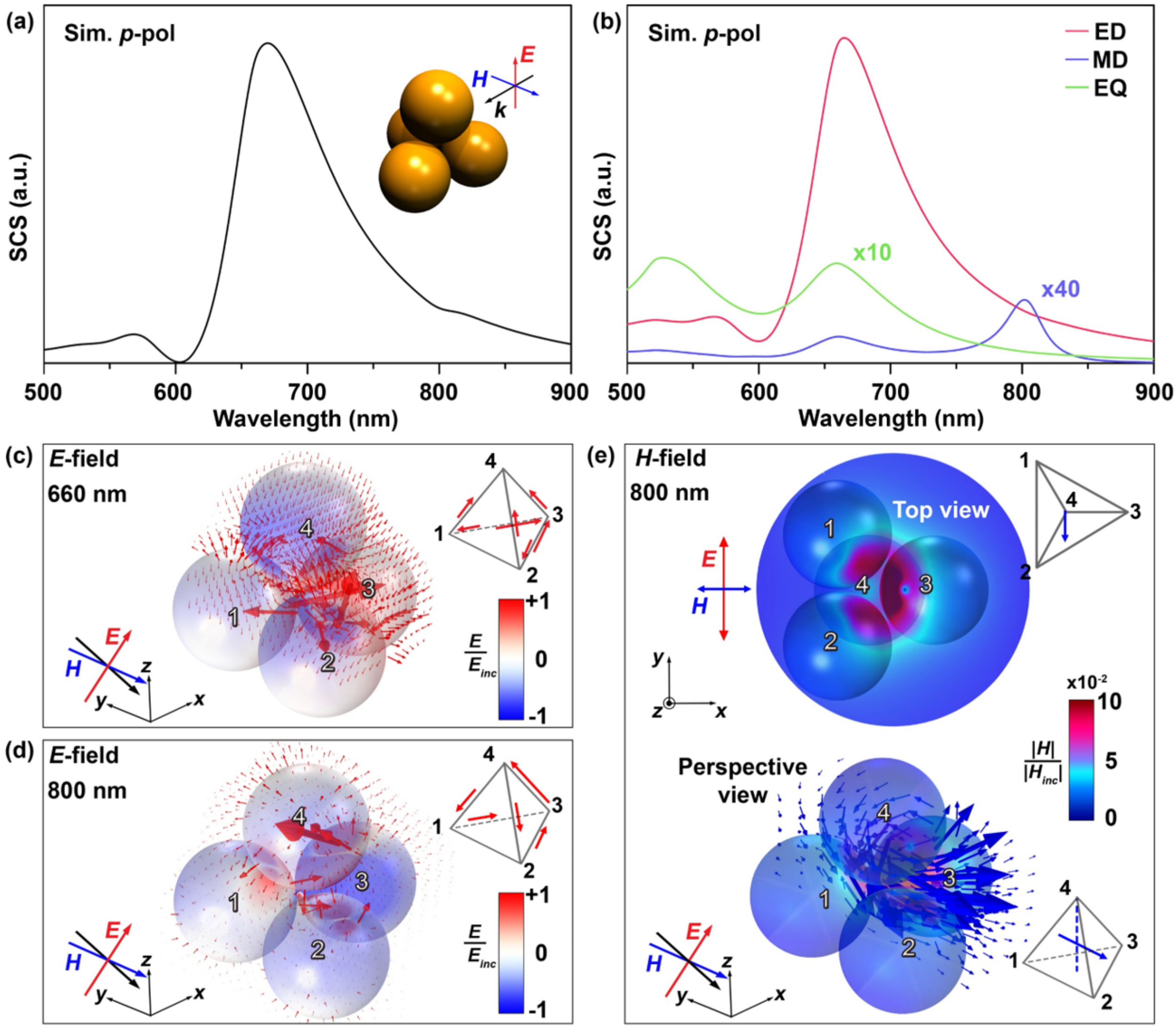

Figure S10. (a-b) Numerically simulated SCS of symmetric tetrahedral cluster for *p*-pol: (a) total SCS and (b) ED/MD/EQ SCS. (c) Spatial distribution of electric field at ED resonance (660 nm) for *p*-pol. (d-e) Spatial distribution of electric field and magnetic field intensity at 800 nm for *p*-pol.

7. Optical properties of asymmetric tetrahedral cluster

4th AuNS can be put onto one of bottom AuNSs rather than the centroid of the symmetric trimer, as presented in Figure S11a-b. Figures S11c-d respectively present theoretical SCS and dark-field scattering spectra of this asymmetric tetrahedral cluster. Theory and experimental results show good agreement. The fundamental ED resonance was divided into two different modes at 660 nm (mode i) and 745 nm (mode ii). The spatial distributions of the induced electric field at these two ED modes are described in Figures S11e-f. Also, it's important to note that out-of-plane loop of the circulating electric field cannot be formed. As a result, the circulating displacement current and electric field was induced only at the bottom trimer (810 nm) (see Figure S11g); due to its weakness, the scattering shoulder was not visible in the total SCS (Figure S11c).

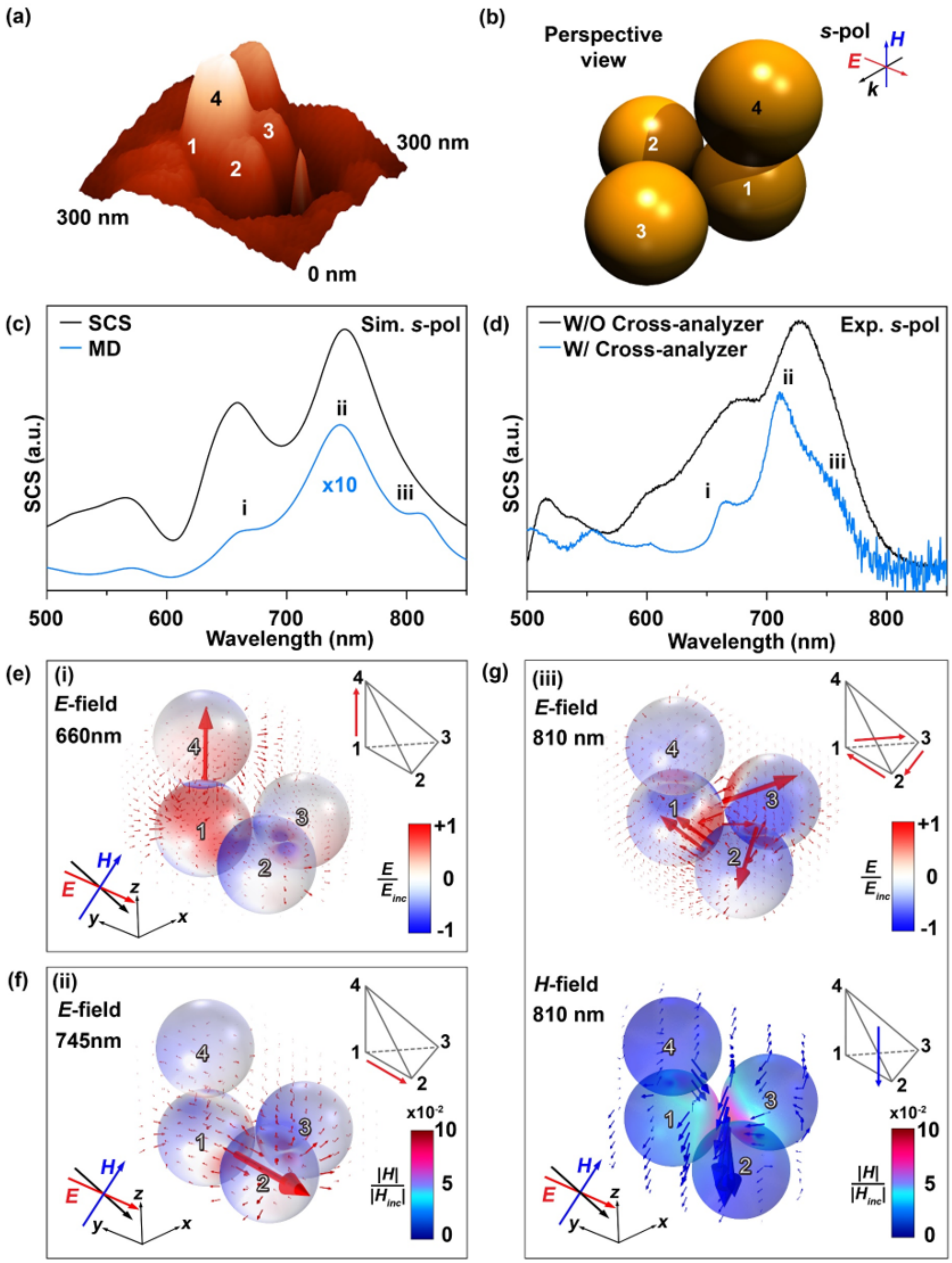

Figure S11. (a-b) 3D AFM image and schematic of asymmetric tetrahedral cluster, assembled by putting 4th AuNS onto the center of 1st AuNS. (c) Numerically simulated total SCS (black line) and MD SCS (light blue line) for *s*-pol. (d) Dark-field scattering spectrum with (black line) and without (light blue line) cross-analyzer for *s*-pol. (e) Spatial distribution of electric field at mode (i) (660 nm). (d) Spatial distribution of electric field at mode (ii) (745 nm). (e) Spatial distribution of electric field (top panel) and magnetic field intensity (bottom panel) at mode (iii).

References

1. Choi, S.-J., Yoo, P. J., Baek, S. J., Kim, T. W., Lee, H. H. An Ultraviolet-Curable Mold for Sub-100-nm Lithography. *J. Am. Chem. Soc.* **126**, 7744−7745 (2004).

2. Laramy, C. R., Brown, K. A., O'Brien, M. N., Mirkin, C. A. High-Throughput, Algorithmic Determination of Nanoparticle Structure from Electron Microscopy Images. *ACS Nano* **9**, 12488–12495 (2015).

3. Perrault, S. D., Chan, W. C. W. Synthesis and Surface Modification of Highly Monodisperse, Spherical Gold Nanoparticles of 50–200 nm. *J. Am. Chem. Soc.* **131**, 17042–17043 (2009).
